# Availability of Grocery Delivery to Food Deserts in States Participating in the Online Purchase Pilot

**DOI:** 10.1001/jamanetworkopen.2019.16444

**Published:** 2019-12-02

**Authors:** Eric J. Brandt, David M. Silvestri, Jerold R. Mande, Margaret L. Holland, Joseph S. Ross

**Affiliations:** 1 National Clinician Scholars Program, Yale University School of Medicine, New Haven, Connecticut; 2 Section of Cardiovascular Medicine, Department of Internal Medicine, Yale University School of Medicine, New Haven, Connecticut; 3 Department of Emergency Medicine, Yale University School of Medicine, New Haven, Connecticut; 4 Friedman School of Nutrition Science and Policy, Tufts University, Boston, Massachusetts; 5 Yale School of Nursing, Yale University, Orange, Connecticut; 6 Section of General Medicine, Department of Internal Medicine, Yale University School of Medicine, New Haven, Connecticut; 7 Department of Health Policy and Management, Yale School of Public Health, New Haven, Connecticut; 8 Center for Outcomes Research and Evaluation, Yale-New Haven Health System, New Haven, Connecticut

## Abstract

This cross-sectional study examines the availability of grocery delivery in census tracts designated as food deserts and among households within them.

## Introduction

The US Department of Agriculture (USDA) Supplemental Nutrition Assistance Program (SNAP) provides federally funded nutritional support to qualifying low-income persons.^1^ To increase SNAP beneficiaries’ access to grocers, the 2014 Farm Bill included an Online Purchase Pilot (OPP) that allows beneficiaries in 8 states to use SNAP to purchase groceries online. The 2018 Farm Bill extends this benefit nationwide after OPP completion. Delivery of online-purchased groceries offers an opportunity to expand food access where it is otherwise limited. However, to our knowledge, there are no studies assessing the availability of grocery delivery in USDA-designated food deserts, defined as low-income communities with limited vehicular access and grocer availability. To inform on the potential effect of delivery in these areas, we quantified the proportion of both rural and urban USDA-designated food deserts currently serviceable by online grocery purchase and delivery in the 8 OPP states.

## Methods

This study was deemed exempt from institutional review board review by Yale University. Informed consent was not required because this study did not involve human subjects. This study is reported following the Strengthening the Reporting of Observational Studies in Epidemiology (STROBE) reporting guideline.

In this cross-sectional study, we used 2015 USDA Economic Research Service Food Access Research Atlas^2^ data to identify USDA-defined food deserts in OPP states. We converted census tracts to zip codes using US Housing and Urban Development US Postal Service zip Code Crosswalk Files^3^ data from September 2018. We identified retailers accepting SNAP using the Nielsen TD-Linx database. From December 2018 through March 2019, we identified corresponding retailer websites through Google (Alphabet) to determine participation in online purchasing and corresponding delivery areas by zip codes. Outcomes were the proportion of food desert census tracts and corresponding SNAP households classified as fully, partially, or not deliverable, according to whether all, some, or no corresponding zip codes were located within grocery delivery areas. We stratified results by urban vs rural status and state, then we compared strata using Monte Carlo simulations to enable comparisons using Fisher exact test owing to many cases of small cell-counts. Data were analyzed using SAS statistical software version 9 (SAS Institute). *P* values were 2-tailed, and statistical significance was set at less than .05.

## Results

In the 8 OPP states, food deserts composed 1250 of 13 134 total census tracts (9.5%), within which 506 863 of 2 760 482 SNAP households (18.4%) were located. Among 1191 urban food desert census tracts, 1108 census tracts (93.0%) were fully deliverable through online grocery purchase and delivery, 13 census tracts (1.1%) were partially deliverable, and 70 census tracts (5.9%) were not deliverable (Table 1). Among 59 rural food desert census tracts, no census tracts were fully deliverable, 18 census tracts (30.5%) were partially deliverable, and 41 census tracts (69.5%) were not deliverable (Table 2). Results were similar for SNAP households within food deserts: 456 263 urban households (92.9%) were fully deliverable, 6466 urban households (1.3%) were partially deliverable, and 28 472 urban households (5.8%) were not deliverable (Table 1), whereas no rural households were fully deliverable, 6121 rural households (39.1%) were partially deliverable, and 9541 rural households (60.9%) were not deliverable (Table 2).

**Table 1.** Availability of Grocery Delivery Among Urban Food Desert Census Tracts and SNAP Households

Geographic Level	No.	Deliverable, No. (%)			*P* Value^a^
		None	Partially	Fully	
Census tracts	1191	70 (5.9)	13 (1.1)	1108 (93.0)	
State census tracts					
Alabama	161	25 (15.5)	6 (3.7)	130 (80.7)	<.001
Iowa	57	9 (15.8)	0	48 (84.2)	
Maryland	198	13 (6.6)	1 (0.5)	184 (92.9)	
Nebraska	37	6 (16.2)	0	31 (83.8)	
New Jersey	192	0	0	192 (100)	
New York	332	8 (2.4)	5 (1.5)	319 (96.1)	
Oregon	95	5 (5.3)	0	90 (94.7)	
Washington	119	4 (3.4)	1 (0.8)	114 (95.8)	
SNAP households	491 201	28 472 (5.8)	6466 (1.3)	456 263 (92.9)	
State SNAP households					
Alabama	67 467	10 250 (15.2)	2490 (3.7)	54 727 (81.1)	<.001
Iowa	21 372	2929 (13.7)	0	18 443 (86.3)	
Maryland	72 485	5939 (8.2)	332 (0.5)	66 214 (91.3)	
Nebraska	12 030	1349 (11.2)	0	10 681 (88.8)	
New Jersey	55 529	0	0	55 529 (100)	
New York	131 450	3392 (2.6)	3007 (2.3)	125 051 (95.1)	
Oregon	64 210	3026 (4.7)	0	61 184 (95.3)	
Washington	66 658	1587 (2.4)	637 (1.0)	64 434 (96.7)	

Abbreviation: SNAP, Supplemental Nutrition Assistance Program.

^a^ Derived from Fisher exact test, estimated using Monte Carlo simulation.

**Table 2.** Availability of Grocery Delivery Among Rural Food Desert Census Tracts and SNAP Households

Geographic Level	No.	Deliverable, No. (%)			*P* Value^a^
		None	Partially	Fully	
Census tracts	59	41 (69.5)	18 (30.5)	0	
State census tracts					
Alabama	20	16 (80.0)	4 (20.0)	0	.006
Iowa	0	NA	NA	NA	
Maryland	2	2 (100)	0	0	
Nebraska	4	4 (100)	0	0	
New Jersey	0	NA	NA	NA	
New York	11	3 (27.3)	8 (72.7)	0	
Oregon	10	9 (90.0)	1 (10.0)	0	
Washington	12	7 (58.3)	5 (41.7)	0	
SNAP households	15 662	9541 (60.9)	6121 (39.1)	0	
State SNAP households					
Alabama	5717	4940 (86.4)	777 (13.6)	0	<.001
Iowa	0	NA	NA	NA	
Maryland	308	308 (100)	0	0	
Nebraska	299	299 (100)	0	0	
New Jersey	0	NA	NA	NA	
New York	3754	1016 (27.1)	2738 (72.9)	0	
Oregon	2290	1870 (81.7)	420 (18.3)	0	
Washington	3294	1108 (33.6)	2186 (66.4)	0	

Abbreviations: NA, not applicable; SNAP, Supplemental Nutrition Assistance Program.

^a^ Derived from Fisher exact test, estimated using Monte Carlo simulation.

The percentage of urban census tracts that were fully deliverable ranged across states from 80.7% in Alabama to 100% in New Jersey (*P* < .001) and from 81.1% in Alabama to 100% in New Jersey for urban SNAP households (*P* < .001) (Table 1). Iowa and New Jersey had no rural food desert census tracts, and no rural census tracts were fully deliverable. However, the percentage of partially deliverable census tracts ranged from 0% in Maryland and Nebraska to 72.7% in New York (*P* = .006) and the percentage of rural SNAP households that were partially deliverable ranged from 0% in Maryland and Nebraska to 72.9% in New York (*P* < .001) (Table 2).

## Discussion

Among 8 states participating in the USDA’s OPP, online grocery purchasing and delivery services were available to more than 90% of urban food desert census tracts and SNAP households within them, but these services were rarely available in rural food desert census tracts. Our results suggest that existing grocery delivery networks, when combined with online grocery-purchasing, could potentially strengthen access to groceries in many areas where it is most lacking. However, grocery delivery fees are not covered by SNAP and may deter online purchasing.^4^ To help maximize OPP benefits in food desert census tracts, the USDA could consider extending SNAP benefits for both online grocery purchasing (as in the OPP) and delivery, although rural areas may be least affected.

This study has limitations. First, our results pertain to food desert census tracts in OPP states and may not be generalizable elsewhere. Second, we may have undercounted delivery services not connected to brick-and-mortar grocery stores or their websites, thereby underestimating delivery availability. Third, we examined grocery delivery areas and not the types or quality of food available for delivery or covered under SNAP. Online ordering could increase or decrease healthy food choices, depending on how platforms are designed.^5^

Online purchasing provides an opportunity to integrate known incentives and disincentives that promote high-quality food purchasing by SNAP beneficiaries.^6^ Delivery might extend these benefits in areas of highest need. Future studies should evaluate how best to leverage and finance online grocery purchasing and delivery to enhance dietary quality, especially among SNAP recipients.
